# Impact of Early versus Late Referral to Nephrologists on Outcomes of Chronic Kidney Disease Patients in Northern India

**DOI:** 10.1155/2022/4768540

**Published:** 2022-06-01

**Authors:** Manoj Dhanorkar, Narayan Prasad, Ravi Kushwaha, Manas Behera, Dharmendra Bhaduaria, Monika Yaccha, Manas Patel, Anupama Kaul

**Affiliations:** Department of Nephrology, Sanjay Gandhi Postgraduate Institute of Medical Sciences, Lucknow, India

## Abstract

**Background:**

CKD patients are often asymptomatic in the early stages and referred late to nephrologists. Late referred patients carry a poor prognosis. There is a lack of data on outcomes associated with referral patterns in CKD patients from northern India.

**Methods:**

In this observational cohort study, all CKD patients who visited the nephrology OPD of the institute between Nov 1, 2018, and Dec 31, 2020, were classified as early referral (ER) if their first encounter with a nephrologist occurred more than one year before initiation of dialysis and education about dialysis (from a nurse or nephrologist). The remaining others were considered late referrals (LRs). The outcomes impact of early and late referrals was analyzed.

**Results:**

A total of 992 (male 656) CKD patients (ER, *n* = 475 and LR, *n* = 517) were enrolled. Patients referred early were older and diabetic and had higher BMI, better education, occupation, and socioeconomic status as compared to those referred late. The mean eGFR at first contact with the nephrologist was (25.4 ± 11.5 ml/min) in ER and 9.6 ± 5.7 ml/min in the LR group and had a higher comorbidity score. The CKD-MBD parameters, hemoglobin, and nutritional parameters were worse in LR. Only a few patients had AVF, and the majority required emergency dialysis in the LR group. A total of 91 (9.2%) patients died, 17 (1.7% ER and 74 (7.5%) patients in the LR group patients. There was significantly lower survival at 6 months (ER 97.1% vs. LR 89.7%), 12 months (ER 96.4% vs. LR 85.7%), 18 months (ER 96.4% vs. LR 85.7%), and 24 months (ER 96.4% vs. LR 85.7%) in late referral group as compared to early referral group (*P*=0.005).

**Conclusions:**

LR to nephrologists has the risk of the emergency start of dialysis with temporary vascular access and had a higher risk of mortality. The timely referral to the nephrologist in the predialysis stage is associated with better survival and reduced mortality.

## 1. Introduction

Patients with end-stage renal disease (ESRD) have exceedingly high morbidity and mortality than the general population [1]. The lack of symptoms in the initial stages of some forms of chronic kidney disease (CKD), especially chronic tubulointerstitial diseases, is recognized late, and more than 50 percent are diagnosed late in CKD 5 on the first presentation [2]. Optimal treatment of CKD patients includes slowing the progression of native diseases, preventing metabolic disorders, preventing malnutrition, preserving the quality of life, and adequate preparation before initiating renal replacement therapy (RRT).

The treatment strategies include optimal blood pressure control, streamlining the CKD-Mineral bone disorders, and anemia management. It also includes timely vaccination against vaccine-preventable blood-borne infections like hepatitis B diseases and pneumococcal and influenza vaccinations in the early stages of CKD before initiating RRT. The timely creation of an arteriovenous fistula (AVF) allows them for a smooth transition from CKD to renal replacement therapy (RRT) [3]. CKD patients who are referred late are often frail and anemic, have a lower likelihood of hepatitis B immunization, start dialysis without an arteriovenous fistula, have a poorer prognosis, and have higher mortality at dialysis initiation [3].

There are multiple factors responsible for late referral in various studies conducted in numerous countries depending on health infrastructure and trained human resources. Meta-analysis of these studies has shown varied factors accountable for the referral and their outcome. Broadly, there are two categories of factors, patient-related and health system-related factors, influencing the referral [4]. Firstly, the patient-related factors include the patient's age, gender, race, comorbidities, etiologies of kidney diseases, noncompliance, and socioeconomic status. Secondly, the health system-related factors are insurance status, referring physicians, referring centers, physician's rationale, and distance to referral centers. The referral to nephrologists from general physicians and the optimal management during the transition up to renal replacement therapy (RRT) affect the outcomes of the CKD patients.

The data on referral patterns and outcomes for CKD patients in the Northern Indian population are lacking. Therefore, we aimed in the current study at a primary objective to find out the factors affecting the referral of the patients and the effect of early and late referrals on outcomes of the patients on subsequent follow-up.

## 2. Subject and Methods

This was a single-center observational cohort study of CKD patients in the North Indian population who visited the nephrology outpatient clinic of the institute. All adult CKD patients 18 years and above who attended the nephrology outpatient department (OPD) of the institute were enrolled after informed consent. A detailed demographic and past medical history along with the clinical examination and laboratory results were entered in a prestructured proforma. Patients were treated as per standard treatment to retard the progression and smooth transition from CKD to renal replacement therapy as per KDIGO clinical practice guidelines during follow-up. CKD was defined as per the KDIGO definition [5]. Estimated glomerular filtration rate (eGFR) was calculated by the Chronic Kidney Disease Epidemiology Collaboration (CKD-EPI) [6].

The data on patients' related factors age, gender, race, education, occupation status, socioeconomic status, modified Charlson comorbidity index (CCI), body mass index (BMI), and blood pressure were recorded. The patient's education and occupation were noted according to Kuppuswamy's socioeconomic scale and later simplified for statistical evaluation [7]. Hematological parameters routinely performed for CKD evaluation like anemia profile hemoglobin, transferrin saturation, ferritin; CKD-MBD parameters of calcium, phosphorus, alkaline phosphatase, vitamin D, intact parathyroid hormone (iPTH); and lipid profiles were collected at the time of the first contact with the nephrologist at the institute. The modified CCI was calculated for each patient on a scale ranging from 0 to 37, calculated as done in a study by Chae et al. [8].

The patients were followed up every 4 months till the endpoint of the study along with supportive care as per KDIGO guidelines. The mean follow-up period was 16.74 ± 4.31 months with minimum and maximum follow-ups of 2 and 24 months, respectively. The patients were divided according to the timing of referral to the nephrologist elsewhere or the nephrologist of the institute. Patients were classified as early referral (ER) if their first encounter with a nephrologist occurred more than one year before initiation of dialysis and education about dialysis (from a nurse or nephrologist). The remaining others were considered late referral (LR) as described previously by Di Napoli et al. [9]. The impact of early and late referral was analyzed on the subsequent outcomes, the requirement of RRT, and the death of the patients on follow-up. The study was conducted after approval from the ethics committee of the institute.

## 3. Statistical Analysis

The continuous variables were expressed as mean ± standard deviation, and the categorical data are expressed as percentages. The Student's *t*-test was used to compare the mean values between the two groups. The chi-square test was used to compare the categorical values with a parametric distribution of values. Mann-Whitney *U* test was used to compare the nonparametric distribution of categorical values. The multivariate logistic regression analysis was used to determine the factors predicting late referral. Cox regression analysis was used to predict the independent variables associated with mortality of patients. The Kaplan-Meier survival analysis was used to analyze and compare the patient's survival between early and late referrals. The log-rank test was used to compare the survival curve for referral patterns. The statistical analysis was performed using IBM SPSS version 25. Significant differences were defined as *P* less than 0.05.

## 4. Results

**Figure 1:**
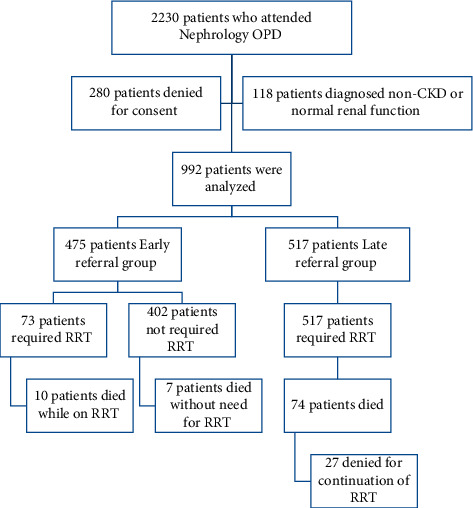
Consort diagram of study flow.

The consort diagram of the study is shown in Figure 1. Two thousand two hundred thirty patients during the first 3 months of enrolment period attended the outpatient department (OPD), of which 1500 patients were referred to as CKD and were considered eligible for inclusion in the study during the study period Nov 1, 2018, to Dec 31, 2020. Two hundred eighty patients did not consent to the study, 118 patients during OPD were diagnosed with a non-CKD disease or normal renal function during follow-up, and 110 patients could not be followed up either physically or telephonically so they were considered ineligible for analysis.

A total of 992 patients (male 656; 72.2%) were analyzed. The gender ratio was similar in both groups. The clinical characteristics of patients with early and late referral patterns are shown in Table 1. Patients referred early were older and had higher BMI, better education, occupation, and socioeconomic status as compared to those referred late. Diabetic patients were referred early as compared to nondiabetic kidney diseases. The systolic and diastolic blood pressures were not different between the groups. The eGFR at first diagnosis with primary physician visits was higher in the early referral group. The early referral group was diagnosed to have CKD at a higher baseline eGFR level and referred at a higher eGFR level to the nephrologist. Patients with late referral had a higher modified Charlson comorbidity score at the time of referral. The mean hemoglobin level was higher in the early group. The iPTH level and serum phosphorus level were high, and serum calcium was low in the late referral group of patients; however, the 25(OH) Vit D was similar. The serum albumin, total cholesterol, and serum triglyceride were lower in the LR group than in the ER group of patients. In the late referral group, 92.5% of patients had elevated serum creatinine, while 84% had elevated serum creatinine at first diagnosis pattern by their primary physician.

The outcome parameters in terms of RRT modality and vascular access concerning early versus late referral are shown in Table 2. At the end of follow-up, a higher proportion of patients (47%) required emergency dialysis (ER 7.5% vs. LR 84%) with a nontunneled catheter (ER 6.1 vs. late 99%) in the late referral group. A significantly higher percentage of patients started on dialysis with AVF as first vascular access in the early referral group (38%) than in the late referral group (0.4%). Patients opting for peritoneal dialysis were not different between the two groups. The number of patients opting for renal transplantation was significantly high in early referral (11%) compared to none in late referral.

The multivariate logistic regression analysis predicting the late referral type is shown in Table 3. The age of the patients, eGFR at the time of diagnosis by primary physician, and modified Charlson comorbidity score were significantly associated with the late referral (Table 3).

## 5. Mortality with Reference to Early and Late Referral Groups

**Figure 2:**
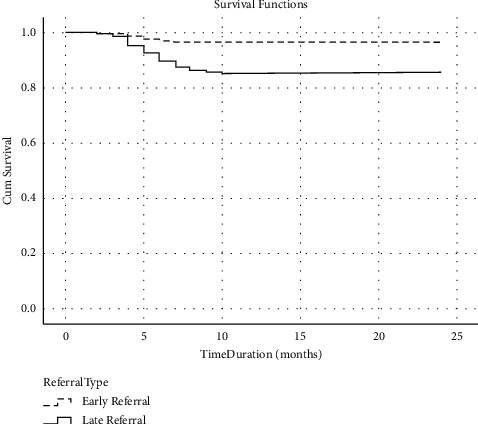
Kaplan-Meier survival curve by the timing of referral pattern in CKD.

The differences in the patient-related characteristics of the dead and alive patients are shown in Table 4. During follow-up, a total of 91 (9.2%) patients died, with 17 (1.7%) in the ER group and 74 (7.5%) patients in the LR group. The relative risk of death of the patients in the LR group (RR 4.31, 95%CI 2.54–7.630) was higher as compared to ER. Besides LR, other factors associated with mortality were age, educational status, eGFR at the time of the first diagnosis by the primary physician, eGFR at the time of referral, number of visits to a nephrologist, and modified Charlson comorbidity score. The hemoglobin level and transferrin saturation were low in patients who died; however, the serum ferritin level was similar. The serum calcium was low, and inorganic phosphorus, alkaline phosphatase, and iPTH values were high in patients who died. The total cholesterol and serum triglyceride were significantly low in those who died. On Kaplan-Meier survival analysis, there was significantly lower survival at 6 months (ER 97.1% vs. LR 89.7%), 12 months (ER 96.4% vs. LR 85.7%), 18 months (ER 96.4% vs. LR 85.7%), and 24 months (ER 96.4% vs. LR 85.7%) in late referral group as compared to early referral group (*P*=0.005) (Figure 2).

The multivariate cox regression analysis predicting the mortality of the patients is shown in Table 5. The age of patients, education, referral type, hemoglobin, calcium, and alkaline phosphatase were the factors significantly associated with the mortality. The LR patients had 2.9 (95% confidence interval 1.27–6.70, *P*=0.012) times higher mortality compared to ER group of patients.

The causes of death in early and late referral groups are enumerated in Table 6. Cardiovascular disease was the most common cause of death (32%), followed by infection (29%) and neoplasm (7%). The remaining deaths (32%) are due to other causes shown in Table 4. In the ER group, cardiovascular disease was the most common cause of death (29%), followed by infection and neoplasm at 23% and 12%, respectively. The remaining deaths (36%) were due to other causes, as shown in Table 6. In the LR group also, the cardiovascular cause was the most common cause of death (32%) followed by infection (30%) and neoplasm (5.4%); however, the death associated with the catheter-related bloodstream infection was significantly higher in the late referral group 23% as compared to no death in early referral group (*P*=0.035).

The outcome characteristics with the modality of RRT and vascular access for dialysis concerning dead and alive patients are shown in Table 7. We also observed significantly higher mortality in patients requiring dialysis (either planned or emergency) with relative risk [9.37 (95% CI 4.28–20.49)] (*P*=0.0001) as compared to patients not requiring dialysis on follow-up. The relative risk of death was high for patients requiring emergency hemodialysis with RR = 3.09 (95% CI 1.92–4.96) (*P*=0.0001) than for patients not requiring dialysis. Patients receiving their first dialysis via nontunneled catheter had significantly higher mortality with RR = 4.75 (95% CI 2.69–8.40) (*P*=0.0001) than other vascular access. Twenty-seven patients in the late referral group who were initiated on emergency HD via nontunneled catheter denied any form of further RRT. All of them died during follow-up, indicating poor acceptance of treatment in the late referral group. Three patients in the ER group underwent timely AVF creation before starting dialysis. Eight patients in the ER group underwent renal transplantation during follow-up compared to none in the late referral group, again reiterating that adequate counseling by the nephrologist is vital for the ideal management of CKD patients.

## 6. Discussion

In this study, we have observed that more than half of the CKD patients had late referral with the first contact with a nephrologist within a year of starting dialysis. We have also observed that diabetic patients with higher education and higher socioeconomic status are referred early. A higher number of patients in the late referral group had an emergency start of dialysis with temporary vascular access, a known risk factor associated with higher mortality in these patients [9–14]. We also observed that CKD patients who were not referred timely to nephrologist die early because of CKD complications. There was a clear survival advantage of the ER groups compared to the LR group on subsequent follow-up as observed in other studies [9–13].

Similar to our study, multiple other studies from the developed and developing countries had also shown higher mortality with the LR. The studies are briefed in Table 8. However, the various studies used different definitions for the ER and LR of CKD patients. The association with the patient outcome also varied in other studies. One study with cut-off timing of late referral of 1 month showed no difference in long term survival; however, a greater financial cost for emergency HD in LR patients was reported [15]. One of the studies with a cut-off duration of 4 months also showed no survival advantage in the long term of early referral; however, authors reported more significant initial morbidity in the late referral group [16].

Most of the latest studies used 12 months to define early referral, which was consistently associated with better outcomes in the early referral group. ER affects predialysis care which includes the creation of access and initiation of RRT. It also helps build the patient and family's financial and mental preparation. ER had better correction of hydration status, various electrolyte imbalances and blood parameters, blood pressure control, evaluation and treatment of comorbidities, etc., which needs a longer duration of preparation of the patient. We used a similar definition of 12-month duration for the categorization of study subjects after enrolment. Fewer patients in ER group required emergency dialysis with the nontunneled catheter. ER patients also had more fistula creation before dialysis. They opted for renal transplantation, again reiterating the fact that adequate counseling by the nephrologist is important for the future prospective management of patients with CKD. The number of AVF creation and patients going for dialysis even in ER groups remained minuscule compared recommended reference [17]. A majority do not opt for any modality of RRT, only a few renal transplantations, and the high death rate suggests no improvement in CKD care and management over the decades [18–20].

Patients with hypocalcemia, high phosphorus, and increased alkaline phosphatase have increased mortality, indicating nonoptimized care for CKD. The low serum albumin, cholesterol, and triglyceride level indicate the poor nutritional status of the patients who died [20]. It also indicates that many of these patients did not receive appropriate supportive care before referral, either due to late diagnosis or due to late referral. Late referral was independently associated with high mortality on multivariate analysis in our study. This indicates a need for the significant role of the primary physician in early diagnosis and referring the CKD patients to a nephrologist for optimum care and smooth transition from early stages of CKD to RRT. Thus, education and sensitization of the primary care physician are equally important.

One of the major strengths of our study was a prospective follow-up of the patients after the first contact with nephrologists. The majority of the studies in the existing pieces of literature had a retrospective observational design (Table 8) with variable timings used for defining the type of referral. This study also has limitations, like a short-term follow-up of patients and a single-center study with referral bias. Furthermore, the referral timing of 3 months seems too short to consider that the patient has received adequate education and counseling before initiating RRT. With a population of 1378 million, India had only 1900 trained nephrologists. It approximates 0.72 nephrologists per million population, far less than the 28 nephrologists per million population in the USA [21, 22]. With limited infrastructure and trained human resources for the optimum care of kidney diseases and RRT, ER for the nephrologists should be made mandatory to optimize the care and intercept preventable death.

In conclusion, LR to nephrologists has the risk of the emergency start of dialysis with temporary vascular access and carries a higher risk of mortality. On the other hand, the timely referral to the nephrologist in the predialysis stage is associated with better survival and reduced mortality in CKD patients.

## Figures and Tables

**Table 1:** Patient-related characteristics and analysis for referral pattern.

	Total (*N* = 992)	ER (*N* = 475) (47.9%)	LR (*N* = 517) (52.1%)	*P* value
Age at the time of referral (yr)	47.6 ± 15.0	51.0 ± 14.3	44.5 ± 15.1	0.005
Gender, male%	656 (72.2%)	343 (72.2%)	373 (72.1%)	0.982
BMI (kg/m^2^)	23.6 ± 4.3	24.3 ± 4.7	23.0 ± 3.9	0.005
Patient's education (%)				
Education below graduate	635 (64)	280 (58.9)	355 (68.7)	0.001
Graduate and postgraduate	357 (36)	195 (41.1)	162 (31.3)	
Patient's occupation (%)				
Primary	631 (63.6)	271 (57.5)	360 (69.1)	0.005
Secondary	172 (17.3)	89 (18.9)	83 (15.9)	
Tertiary	189 (19.1)	111 (23.6)	78 (15)	
Socioeconomic class (%)				
Lower	384 (38.7)	147 (30.9)	237 (45.8)	0.005
Middle	587 (59.2)	314 (66.1)	273 (52.8)	
Upper	21 (2.1)	14 (2.9)	7 (1.4)	
Underlying kidney disease (%)				
Diabetic kidney disease	252 (25.4)	153 (32.2)	99 (19.1)	0.005
Glomerulonephritis	246 (24.8)	100 (21.2)	146 (28.2)	
Chronic tubulointerstitial nephritis	435 (43.9)	187 (39.3)	248 (48)	
Polycystic kidney disease	28 (2.8)	15 (3.1)	13 (2.6)	
Hypertensive renal disease	27 (2.7)	17 (3.6)	10 (1.9)	
Unknown	4 (0.4)	3 (0.6)	1 (0.2)	
Systolic BP (mmHg)	147.6 ± 23.2	147.3 ± 22.6	147.9 ± 23.7	0.684
Diastolic BP (mmHg)	83.8 ± 15.8	83.5 ± 15.2	84.0 ± 16.3	0.572
eGFR at the time of diagnosis by primary physician (ml/min/m^2^)	23.0 ± 15.4	31.5 ± 13.9	15.3 ± 12.2	0.005
Number of visits to nephrologist from referral to dialysis				
2 times or more	581 (58.6)	471 (99.2)	110 (21.3)	0.005
1 time	30 (3)	4 (0.8)	26 (5)	
None	381 (38.4)	0 (0)	381 (73.7)	
Median duration from renal disease diagnosis by primary physician to referral (month) (IQR)	4 (11)	6 (18)	3 (9)	0.005
Median duration of follow-up by primary physician till CKD diagnosis (month) (IQR)	2 (36.8)	1 (13.6)	10 (10)	0.005
Primary physician (%)				
General physician	310 (31.3)	111 (23.4)	199 (38.5)	0.005
Postgraduate physician	682 (68.8)	364 (76.6)	318 (61.5)	
Modified Charlson comorbidity index	3.4 ± 1.5	3.2 ± 1.7	3.5 ± 1.2	0.001
Hemoglobin (g/dL)	9.7 ± 4.0	10.4 ± 2.1	9.0 ± 5.0	0.005
Transferrin saturation (%)	32.4 ± 129.2	25.9 ± 27.3	37.7 ± 171.7	0.182
Sr. ferritin (ng/mL)	385.4 ± 490.0	264.4 ± 385.8	482.2 ± 541.3	0.005
Corrected calcium (mg/dL)	8.7 ± 1.1	9.0 ± 0.9	8.4 ± 1.3	0.005
Phosphorus (mg/dL)	5.9 ± 8.9	4.7 ± 1.3	7.0 ± 2.2	0.005
Alkaline phosphate (IU/L)	139.4 ± 98.3	122.3 ± 67.7	155.0 ± 117.6	0.005
Vit D (nmol/L)	21.3 ± 19.2	21.7 ± 17.5	21.0 ± 20.7	0.631
Median intact PTH (ng/L) (IQR)	369.9 (477.7)	259.2 (293.4)	502.9 (603.9)	0.005
Protein (g/dL)	7.3 ± 4.0	7.6 ± 5.2	7.0 ± 2.6	0.037
Albumin (g/dL)	3.9 ± 0.8	4.0 ± 0.7	3.9 ± 0.1	0.038
Total cholesterol (mg/dL)	173.5 ± 63.3	179.1 ± 65.8	167.6 ± 60.1	0.010
Triglyceride (mg/dL)	152.0 ± 84.3	159.8 ± 92.6	143.8 ± 74.0	0.007
Uric acid (mg/dL)	7.9 ± 4.1	7.8 ± 4.3	8.0 ± 4.0	0.622
Usual presentation at the time of diagnosis (%)				
Elevated serum creatinine	877 (88.4)	399 (84)	478 (92.5)	0.005
Abnormal kidney or urinary tract	66 (6.7)	40 (8.4)	26 (5)	
Urine abnormalities	49 (4.9)	36 (7.6)	13 (2.5)	

**Table 2:** Outcome-related characteristics with reference to referral pattern.

	Total (*N* = 992) (100%)	ER (*N* = 475)	LR (*N* = 517)	*P* value
*RRT initiation type (%)*				
No requirement of RRT	402 (40.5)	402 (84.6)	0 (0)	0.005
Planned RRT	122 (12.3)	38 (8)	84 (16.2)	
Emergency RRT	468 (47.2)	35 (7.4)	433 (83.8)	
*First dialysis access (%)*				
No	402 (40.5)	402 (84.6)	0 (0)	0.005
Nontunneled catheter	541 (54.5)	29 (6.1)	512 (99)	
Tunneled catheter	19 (2)	16 (3.4)	3 (0.6)	
Fistula	30 (3)	28 (5.9)	2 (0.4)	
*Current dialysis access at end of follow-up*				
No on RRT	426 (42.9)	399^#^ (84)	27^*∗*^ (5.2)	0.005
Nontunneled catheter	38 (3.8)	0 (0)	38 (7.4)	
Tunneled catheter	156 (15.7)	21 (4.5)	135 (26.1)	
Fistula	363 (36.7)	51^#^ (10.7)	312 (60.3)	
CAPD/APD	9 (0.9)	4 (0.8)	5 (1)	
*RRT modality in follow-up (%)*				
None	429 (43.3)	402 (84.7)	27^*∗*^ (5.2)	0.005
Hemodialysis	546 (55)	61 (12.8)	485 (93.8)	
Peritoneal dialysis	9 (0.9)	4 (0.8)	5 (1)	
Renal transplant	8 (0.8)	8^$^ (1.7)	0 (0)	

^*∗*^27 patients in LR group denied any RRT type on follow-up and hence had no final dialysis access and all died during follow-up. ^#^3 patients in ER group made fistula in follow-up but did not require any RRT till the final follow-up. ^$^8 patients who were initially on HD later underwent renal transplant. RRT, renal replacement therapy.

**Table 3:** Multivariate analysis showing variables associated with late referral.

Variable	HR	95% CI	*P* value
Age at the time of referral (per year)	1.02	1.01–1.04	0.005
Body mass index (per kg/m^2^)	1.03	0.99–1.07	0.108
Patient's education, education below graduate (ref)			
Graduate and postgraduate	0.84	0.56–1.26	0.388
Patient's occupation, primary (ref)			
Secondary	1.19	0.71–1.99	0.521
Tertiary	0.83	0.46–1.48	0.533
Socioeconomic class, lower (ref)			
Middle	0.86	0.27–2.77	0.804
Upper	1.27	0.42–3.85	0.668
eGFR at the time of diagnosis by primary physician (ml/min/m^2^)	1.10	1.08–1.11	0.005
Duration of follow-up by primary physician till CKD diagnosis (per month)	1.00	1.00–1.01	0.095
Duration of follow-up from renal disease diagnosis by primary physician to referral (per month)	1.00	1.00 – 1.00	0.106
Primary physician, general physician (ref)			
Postgraduate physician	0.95	0.66–1.37	0.793
Modified Charlson comorbidity index	0.72	0.64–0.82	0.005
Underlying kidney disease, diabetic kidney disease (ref)			
CKD other than DKD	0.94	0.62–1.42	0.760

CKD, chronic kidney disease; DKD, diabetic kidney disease; eGFR, glomerular filtration rate.

**Table 4:** Differences in clinical parameters between alive and dead patients on follow-up.

	Total (*N* = 992) (100%)	Death (*N* = 91) (9.2%)	Alive (*N* = 901) (90.8%)	*P*-value
Age at the time of referral (yr)	47.6 ± 15.0	55.5 ± 15.1	47.1 ± 15.0	**0.001**
Gender, male%	716 (72.2)	61 (6.2)	655 (66)	0.251
BMI (kg/m^2^)	23.6 ± 4.3	23.5 ± 4.0	23.6 ± 4.4	0.845
Patient's education (%)				
Education below graduate	635 (64)	68 (74.7)	567 (62.9)	**0.025**
Graduate and postgraduate	357 (36)	23 (25.3)	334 (37.1)	
Patient's occupation (%)				
Primary	631 (63.6)	66 (72.5)	565 (62.7)	0.179
Secondary	172 (17.3)	12 (13.2)	160 17.8)	
Tertiary	189 (19.1)	13 (14.3)	176 (19.5)	
Socioeconomic class (%)				
Lower	384 (38.7)	43 (47.3)	341 (37.8)	0.126
Middle	587 (59.2)	45 (49.1)	542 (60.2)	
Upper	21 (2.1)	3 (3.3)	18 (2.0)	
Referral type				
Late referral	521 (52.5)	74 (81.3)	447 (49.6)	**0.005**
Early referral	471 (47.5)	17 (18.7)	454 (50.4)	
Underlying kidney disease (%)				
Diabetic kidney disease	252 (25.4)	20 (22)	232 (25.7)	0.431
CKD other than DKD	740 (74.6)	71 (78)	669 (74.3)	
Systolic BP (mmHg)	147.6 ± 23.2	149.5 ± 22.1	147.4 ± 23.3	0.414
Diastolic BP (mmHg)	83.8 ± 15.8	81.5 ± 15.2	84.0 ± 15.8	0.148
eGFR at the time of diagnosis by primary physician (ml/min/m^2^)	23.0 ± 15.4	16.9 ± 12.1	23.7 ± 15.5	**0.005**
Number of visits to nephrologist from referral to dialysis				
2 times or more	585 (59)	32 (35.2)	553 (61.4)	**0.005**
1 time	26 (2.6)	4 (4.4)	22 (2.4)	
None	381 (38.4))	55 (60.4)	326 (36.2)	
Median duration from renal disease diagnosis by primary physician to referral (mo) (IQR)	4 (11)	5 (15.5)	4 (11)	0.262
Median duration of follow-up by primary physician till CKD diagnosis (mo) (IQR)	2 (36.8)	2 (23)	3 (37)	0.213
Primary physician (%)				
General physician	310 (31.3)	33 (36.3)	277 (30.7)	0.279
Postgraduate physician	682 (68.7)	58 (63.7)	624 (69.3)	
Modified Charlson comorbidity index	3.4 ± 1.5	3.9 ± 1.5	3.3 ± 1.4	**0.005**
Hemoglobin (g/dL)	9.7 ± 4.0	8.4 ± 2.0	9.8 ± 4.1	**<0.005**
Transferrin saturation (%)	32.5 ± 129.2	26.2 ± 15.5	33.1 ± 135.6	0.647
Sr. ferritin (ng/mL)	385.4 ± 490.0	452.5 ± 550.7	378.6 ± 483.3	0.647
Corrected calcium (mg/dL)	8.7 ± 1.1	8.1 ± 1.1	8.8 ± 1.1	**0.005**
Phosphorus (mg/dL)	5.9 ± 8.9	6.8 ± 2.1	5.8 ± 9.3	**0.005**
Alkaline phosphate (IU/L)	139.4 ± 98.3	173.5 ± 133.4	135 ± 93.4	**0.001**
Vit D (nmol/L)	21.3 ± 19.2	18.3 ± 15.3	21.6 ± 19.5	0.229
Median intact PTH (ng/L) (IQR)	370 (477.7)	559 (548)	346 (442)	**0.005**
Protein (g/dL)	7.3 ± 4.0	7.4 ± 5.8	7.3 ± 3.8	0.679
Albumin (g/dL)	3.9 ± 0.8	3.8 ± 1.7	3.9 ± 0.6	0.215
Total cholesterol (mg/dL)	173.5 ± 63.3	157.5 ± 69.8	175.0 ± 62.4	**0.027**
Triglyceride (mg/dL)	152.0 ± 84.3	125.9 ± 71.5	154.5 ± 85.1	**0.007**
Serum sodium (mEq/L)	136.5 ± 5.1	135.5 ± 5.8	136.6 ± 5.1	**0.049**
Serum potassium (mEq/L)	5.0 ± 0.8	5.1 ± 0.8	5.0 ± 0.8	0.224
Serum uric acid (mg/dL)	7.9 ± 4.1	8.0 ± 2.3	7.9 ± 4.3	0.843
Usual presentation for first kidney disease diagnosis (%)				
Elevated serum creatinine	877 (88.4)	85 (93.4)	792 (87.9)	**0.176**
Abnormal kidney or urinary tract	66 (6.7)	5 (5.5)	61 (6.8)	
Urine abnormalities	49 (4.9)	1 (1.1)	48 (5.3)	

**Table 5:** Multivariate cox regression analysis showing independent variables associated with mortality.

Multivariate cox regression analysis of predictors associated with mortality			
Age at the time of referral (per year increase)	1.05	1.03–1.07	**0.005**
*Patient's education, education below graduate (ref)*			
Graduate and postgraduate	0.47	0.24–0.92	**0.027**
eGFR at first diagnosis by primary physician (per ml/min/m^2^)	0.99	0.96–1.01	0.299

*Referral type, early referral (ref)*			
Late referral	2.91	1.27–6.70	**0.012**
Modified Charlson comorbidity index	1.13	0.93–1.37	0.235

*Number of visits to nephrologist from referral to dialysis, none (reference)*			
1 time	0.85	0.42–1.72	0.642
2 times or more	0.73	0.16–3.40	0.685
Hemoglobin (g/dL)	0.85	0.73–0.99	**0.034**
Serum calcium (mg/dL)	0.68	0.53–0.88	**0.003**
Phosphorus (mg/dL)	1.01	1.00–1.02	0.265
Alkaline phosphate (IU/L)	1.00	1.00–1.00	**0.026**
Vit D (nmol/L)	0.99	0.97 – 1.01	0.218
Intact PTH (ng/L)	1.00	1.00–1.00	0.622
Total cholesterol (mg/dL)	1.00	1.00–1.01	0.662
Triglyceride (mg/dL)	1.00	0.99–1.00	0.237

**Table 6:** Cause of death in patients on follow-up in early and late referral group.

	Cause of death	Early referral (*n* = 17)	Late referral (*n* = 74)	Total (*n* = 91)
Cardiovascular disease	Myocardial infarction	0	2	2
	Cardiomyopathy	0	1	1
	Cardiac arrest, cause unknown	4	14	18
	Pulmonary edema	0	2	2
	Pulmonary embolus	0	1	1
	Cerebrovascular accidents including intracranial hemorrhage	1	3	4
	Other hemorrhage	0	1	1

Infections	Catheter-related blood stream infection	0	17	17
	Peritoneal access infection complication	2	0	2
	Septicemia, other causes	0	1	1
	Pulmonary infections (pneumonia, pyothorax)	2	3	5
	Endocarditis	0	1	1

Liver and abdominal disease	Liver failure	1	3	4
Neoplasm	Metastatic disease/solid tumor	1	1	2
	Multiple myeloma	1	3	4

Other	Hyperkalemia	2	5	7
	Severe cachexia/failure to thrive	0	2	2
	Opportunistic infection	1	2	3
	Suicide	0	1	1
	Another cause of death	1	1	2
	Unknown	1	10	11

**Table 7:** Differences in outcome-related characteristics regarding dead and alive patients on follow-up.

	Total (*N* = 992) (100%)	Death (*N* = 91) (9.2%)	Alive (*N* = 901) (90.8%)	*P* value
*RRT initiation type (%)*				
No requirement of RRT	402 (40.5)	7^#^ (7.7)	395 (43.8)	<0.005
Planned RRT	122 (12.3)	19 (20.9)	103 (11.4)	
Emergency RRT	468 (47.2)	65 (71.4)	403 (44.7)	
*First dialysis access (%)*				
No	402 (40.5)	7^#^ (7.7)	395 (43.8)	<0.005
Nontunneled catheter	541 (54.5)	76 (83.5)	465 (51.6)	
Tunneled catheter	19 (1.9)	3 (3.3)	16 (1.8)	
Fistula	30 (3.1)	5 (5.5)	25 (2.8)	
*Current dialysis access at end of follow-up*				
No on RRT	426 (42.9)	34^*∗*^ (37.3)	392 (43.5)	<0.005
Nontunneled catheter	38 (3.8)	15 (16.5)	23 (2.6)	
Tunneled catheter	156 (15.8)	22 (24.2)	134 (14.9)	
Fistula	363 (36.6)	18 (19.8)	345 (38.2)	
CAPD/APD	9 (0.9)	2 (2.2)	7 (0.8)	
*RRT modality in follow-up (%)*				
None	429 (43.2)	34^*∗*^ (37.4)	395^@^ (43.8)	0.264
Hemodialysis	546 (55.1)	55 (60.4))	491 (54.5)	
Peritoneal dialysis	9 (0.9)	2 (2.2)	7 (0.8)	
Renal transplant	8 (0.8)	0 (0)	8^$^ (0.9)	

^*∗*^27 patients in the LR group denied any RRT type in follow-up and all died during follow-up. ^#^7 patients in ER group died without the requirement of RRT. ^@^3 patients made fistula in follow-up but did not require any RRT till the final follow-up. ^$^8 patients who were initially on HD later underwent renal transplant.

**Table 8:** Summary of the studies with outcomes in the early versus late referral.

Study	ER/LR definition	Outcome
Kazmi et al. 2004, 2,195 patients; USRDS [12]	Late <4 months	44% higher mortality in LR group
	Early >4 months	

Dogan et al. 2005, 101 patients; Turkey [11]	Late <12 weeks	Better biochemical variables, short hospital stay, higher AVF creation, and availability to start alternative dialysis modality (CAPD)
	Early >12 weeks	

De Jager et al. 2010, 1438 patients; Netherland [10]	Late <3 months	Early and late referrals were associated with increased mortality compared with very early referral
	Early (3–12 months)	
	Very early (>12 months)	

Kim et al. 2013, 1028 patients; Korea [13]	Late <12 months	Reduced morbidity and mortality and hospitalization, better uptake of PD and AV fistula creation in the ER group
	Early >12 months	

Di Napoli et al. 2010 673 patients; Italy [9]	Late <12 months	Lower frequency of hepatitis B virus vaccination, arteriovenous fistula, and information about renal replacement therapy modalities, emergency initiation of HD in LR group
	Early >12 months	

Schmidt et al. 1998, 238 patients, United States [15]	ER > 1 month	No difference in long term survival but greater financial cost for emergency HD in LR patients
	LR < 1 month	

Roubicek et al. 2000 270 patients, France [16]	ER > 4 months	Greater initial morbidity in late referral group but no difference in long term outcome
	LR < 4 months	

## Data Availability

The data are available with the first authors and corresponding author and can be made available upon reasonable request. However, they are not made public due to ethical and regulatory issues.
